# Multiple Genes of Symbiotic Plasmid and Chromosome in Type II Peanut *Bradyrhizobium* Strains Corresponding to the Incompatible Symbiosis With *Vigna radiata*

**DOI:** 10.3389/fmicb.2020.01175

**Published:** 2020-06-23

**Authors:** Yue Wu, Yong Hua Li, Jiao Ying Shang, En Tao Wang, La Chen, Bin Huo, Xin Hua Sui, Chang Fu Tian, Wen Feng Chen, Wen Xin Chen

**Affiliations:** ^1^State Key Laboratory for Agrobiotechnology, Key Laboratory of Soil Microbiology, Ministry of Agriculture, College of Biological Sciences, China Agricultural University, Beijing, China; ^2^Escuela Nacional de Ciencias Biologicas, Instituto Politecnico Nacional, Mexico City, Mexico

**Keywords:** *Bradyrhizobium*, *Vigna radiata*, incompatible symbiosis, interactions, genetic differences, protein 3D structure

## Abstract

Rhizobia are capable of establishing compatible symbiosis with their hosts of origin and plants in the cross-nodulation group that the hosts of origin belonged to. However, different from the normal peanut *Bradyrhizobium* (Type I strains), the Type II strains showed incompatible symbiosis with *Vigna radiata*. Here, we employed transposon mutagenesis to identify the genetic loci related to this incompatibility in Type II strain CCBAU 53363. As results, seven Tn*5* transposon insertion mutants resulted in an increase in nodule number on *V. radiata*. By sequencing analysis of the sequence flanking Tn*5* insertion, six mutants were located in the chromosome of CCBAU 53363, respectively encoding acyltransferase (L265) and hypothetical protein (L615)—unique to CCBAU 53363, two hypothetical proteins (L4 and L82), tripartite tricarboxylate transporter substrate binding protein (L373), and sulfur oxidation c-type cytochrome SoxA (L646), while one mutant was in symbiotic plasmid encoding alanine dehydrogenase (L147). Significant differences were observed in L147 gene sequences and the deduced protein 3D structures between the Type II (in symbiotic plasmid) and Type I strains (in chromosome). Conversely, strains in both types shared high homologies in the chromosome genes L373 and L646 and in their protein 3D structures. These data indicated that the symbiotic plasmid gene in Type II strains might have directly affected their symbiosis incompatibility, whereas the chromosome genes might be indirectly involved in this process by regulating the plasmid symbiosis genes. The seven genes may initially explain the complication associated with symbiotic incompatibility.

## Introduction

Symbiotic relationships between legume plants and soil bacteria, collectively termed rhizobia, are characterized by the formation of root nodules, a specialized plant organ, in which rhizobia differentiate into nitrogen-fixing bacteroids and reduce nitrogen to ammonia as nutrient for plant. In exchange, plants provide specialized environment and carbohydrates to rhizobia (Krishnan et al., 2003; Nguyen et al., 2017). The association between legumes and rhizobia is highly specific, meaning that each rhizobial species establishes symbiosis with only a limited set of host plants and vice versa; this specificity led to the definition of cross-nodulation groups, which is used for description of symbiotic diversity and rhizobial species (Yang et al., 2010). The symbiotic specificity is determined by a fine-tuned exchange of molecular signals between host plant and its bacterial symbiont (Perret et al., 2000). Rhizobial specificity-related factors, such as NodD, exopolysaccharides, lipopolysaccharides, secreted proteins, Nod-factors, and so on, have been reported to affect the nodulation and host specificity (Radutoiu et al., 2007; Okazaki et al., 2013). Mutations in these related genes can cause incompatible symbiosis between rhizobia and legumes with the phenomenon that a rhizobium is unable to nodulate a particular host plant or forms nodules that are incapable of fixing nitrogen (Faruque et al., 2015; Wang et al., 2018). This incompatible relationship takes place at the early stages of the interaction and is demonstrated to result from signal changing between the host plants and bacteria, which is the molecular basis for the recognition mechanisms evolved in the process of coadaptation (Tang et al., 2016; Fan et al., 2017). This phenomenon also frequently happens at the later stages of nodule development with causing nitrogen-fixing efficiency difference between various plant–bacterium combinations (Wang et al., 2017; Yang et al., 2017). Studies have shown that genes with different functions participate in the control of incompatible symbiosis between rhizobia and plants. In *Bradyrhizobium elkanii* USDA 61, T3 secretion system (T3SS) participates in its incompatible symbiosis with *Vigna radiata* plant (Nguyen et al., 2017). In *Bradyrhizobium diazoefficiens* USDA 110, the metabolic pathways, transporters, chemotaxis, and mobility negatively influence the nodulation with *Glycine max* (host of origin) and *Sophora flavescens* (incompatible host) (Liu et al., 2018a). Cell surface exopolysaccharides (EPS) in *Sinorhizobium meliloti* and lipopolysaccharide (LPS) in *Mesorhizobium loti* 2231 were reported to affect the incompatible symbiosis with *Medicago sativa* (Barnett and Long, 2018) and *Lotus corniculatus* (Turska-Szewczuk et al., 2008), respectively.

It is generally believed that peanut (*Arachis hypogaea* L.) and mung bean (*V. radiata*) belong to the same cross-nodulation group; therefore, the peanut bradyrhizobia have the ability of establishing effective symbiosis with *V*. *radiata* (Zhang et al., 2011; Li et al., 2019). However, our previous study revealed that the majority of peanut bradyrhizobia (Type I) could establish normal symbiosis with *V*. *radiata* and the minority of strains (Type II) showed incompatible symbiosis with the same plant, and all the Type II strains contained a symbiotic plasmid (Li, 2019). In detail, Type I strains formed efficient and numerous nodules, and Type II strains formed ineffective and less nodules with *V. radiata* (Li, 2019). Genotype-specific symbiotic compatibility in interactions between legumes and rhizobia is an important trait for the use of root nodule bacteria to improve the crop yield (Triplett and Sadowsky, 1992). The incompatible symbiosis between the peanut rhizobia and *V. radiata* offered a valuable model for investigation of the mechanisms involved in the symbiotic efficiency of rhizobia, which is not clearly described up to date.

In order to understand the causes for the incompatible interaction between Type II strains and *V. radiata*, we performed the present study. A genetic approach of Tn*5* transposon mutagenesis was taken with Type II representative strain *B. guangxiense* CCBAU 53363 to construct a mutant library for screening the potential genes that regulate its effective nodulation on *V. radiata* plant. The mutants with compatible symbiotic phenotype with *V. radiata* were selected by nodulation experiments. Mutational analysis identified seven genes associated with the symbiotic incompatibility, and subsequently, the 3D structures of their predicted proteins were compared between the Type I and II strains. The results in this study would improve our understanding about the symbiotic incompatible mechanisms in legume–rhizobium interactions.

## Materials and Methods

### Bacterial Strains and Growth Conditions

Bacterial strains, plasmids, and primers used in this study are listed in Table 1 and Supplementary Table S1. Rhizobia and *Escherichia coli* strains were cultured with tryptone yeast (TY) or yeast mannitol agar (YMA) medium at 28°C (Beringer, 1974) and Luria–Bertani (LB) medium at 37°C (Sambrook et al., 1989), respectively. When required, the media were supplemented with sucrose (7%, wt/vol) and/or antibiotics at the final concentrations of kanamycin (Km), 50 μg/ml; gentamicin (Gen), 30 μg/ml; and trimethoprim (Tmp), 10 μg/ml.

**TABLE 1:** Bacterial strains and plasmids used in this study.

**Strains or plasmids**	**Genotype or relevant characteristics**	**Source or references**
**Bacterial strains**		
*Bradyrhizobium guangxiense*		
CCBAU 53363	Wild type of Type II strain; Tmp^r^	Li et al., 2015
L4-T	Tn*5* inserted in gene encoding for hypothetical protein, Tmp^r^, Km^r^	This work
L4-P	pJQ200SK knockout in gene encoding for hypothetical protein, Tmp^r^	This work
L82-T	Tn*5* inserted in gene encoding for hypothetical protein, Tmp^r^, Km^r^	This work
L82-P	pJQ200SK knockout in gene encoding for hypothetical protein, Tmp^r^	This work
L147-T	Tn*5* inserted in *ald* gene encoding for alanine dehydrogenase, Tmp^r^, Km^r^	This work
L147-P	pJQ200SK knockout in *ald* gene encoding for alanine dehydrogenase, Tmp^r^	This work
L265-T	Tn*5* inserted in gene encoding for acyltransferase, Tmp^r^, Km^r^	This work
L265-P	pJQ200SK knockout in gene encoding for acyltransferase, Tmp^r^	This work
L373-T	Tn*5* inserted in gene encoding for tripartite tricarboxylate transporter substrate binding protein, Tmp^r^, Km^r^	This work
L373-P	pJQ200SK knockout in gene encoding for tripartite tricarboxylate transporter substrate binding protein, Tmp^r^	This work
L615-T	Tn*5* inserted in gene encoding for hypothetical protein, Tmp^r^, Km^r^	This work
L615-P	pJQ200SK knockout in gene encoding for hypothetical protein, Tmp^r^	This work
L646-T	Tn*5* inserted in *soxA* gene encoding for sulfur oxidation c-type cytochrome SoxA, Tmp^r^, Km^r^	This work
L646-P	pJQ200SK knockout in gene encoding for sulfur oxidation c-type cytochrome SoxA, Tmp^r^	This work
*B. guangzhouense*		
CCBAU 51670^T^	Type II strain; Tmp^r^	Li et al., 2019
*B. guangdongense*		
CCBAU 51649^T^	Type II strain; Tmp^r^	Li et al., 2019
*B. nanningense*		
CCBAU 53390^T^	Type I strain; Tmp^r^	Li et al., 2019
CCBAU 51757	Type I strain; Tmp^r^	Li et al., 2019
*B. zhanjiangense*		
CCBAU 51787	Type I strain; Tmp^r^	Li et al., 2019
CCBAU 51778^T^	Type I strain; Tmp^r^	Li et al., 2019
*E. coli*		
DH5α	*supE44*△*lacU169* (∮80*lacZ*△M15) *hsdR17 recA1 endA1 gyrA96 thi-1 relA1*	Sambrook et al., 1989
**Plasmids**		
pRK2013	Helper plasmid that provides plasmid transfer functions; Km^r^	Figurski and Helinski, 1979
pRL1063a-2	Suicide plasmid for Tn*5* mutagenesis of *Bradyrhizobium*, containing *sacB* gene (sucrose sensitive gene) from pJQ200SK; Km^r^	This work
pJQ200SK	*sacB*, suicide plasmid for mutagenesis of *Bradyrhizobium*; Gen^r^	Quandt and Hynes, 1993
pJQ200SK-L4	Constructed plasmid for L4 gene knockout	This work
pJQ200SK-L82	Constructed plasmid for L82 gene knockout	This work
pJQ200SK-L147	Constructed plasmid for L147 gene knockout	This work
pJQ200SK-L265	Constructed plasmid for L265 gene knockout	This work
pJQ200SK-L373	Constructed plasmid for L373 gene knockout	This work
pJQ200SK-L615	Constructed plasmid for L615 gene knockout	This work
pJQ200SK-L646	Constructed plasmid for L646 gene knockout	This work
**Primers**	Sequences	
415L	5′-CCATTTGCCACTCTCCTTT-3′ (7,247–7,265 bp)	Liu et al., 2018b
415R	5′-TACTGCCCGCTTGGTTAA-3′ (7,644–7,661 bp)	Liu et al., 2018b
T5BF	5′-TTGCTCGTCGGTGATGTA-3′ (10,549–10,566 bp)	This work
T5BR	5′-TGCCAAAGGGTTCGTGTA-3′ (11,210–11,227 bp)	This work
PM	5′-TCATCTAATGCTAAGGCTGC-3′ (199–218 bp)	Liu et al., 2018b

### Tn*5* Mutant Library and Positive Clones Screening

A Tn*5* insertion mutant library of CCBAU 53363 was built by triparental conjugation method reported by Liu et al. (2018b) with some modifications. Tn*5* transposon was introduced into CCBAU 53363 (recipient) by conjugative transfer of the plasmid pRL1063a-2 (donor) with the help of plasmid pRK2013 (helper). Due to the low Tn*5* transposition efficiency (1.25%), using pRL1063a plasmid as donor in CCBAU 53363 chromosome, pRL1063a-2 was constructed in this study by inserting the *sacB* gene (sucrose sensitive gene) in the *Eco*RI site of pRL1063a by seamless cloning, which was located downstream of the Tn*5* transposon gene of pRL1063a (see plasmid structure in Wolk et al., 1991). Then, the plasmid pRL1063a-2 was used as the donor in triparental conjugate test, and the transposition efficiency was significantly increased to 18%. After 4 days’ triparental conjugating, transconjugants were selected on TY medium containing Tmp, Km, and sucrose. Colonies grown on plates were collected and washed with 0.8% of NaCl solution, resuspended to the concentration of OD_600_ = 0.2, and inoculated to *V. radiata* seedlings at the dose of 1 ml/plant. A total of 400 plants were grown in Leonard jars filled with vermiculite moistened with low-N nutrient solution (Vincent, 1970) at 25°C in greenhouse with a daylight illumination period of 12 h. Nodules were harvested in 30 days postinoculation (dpi) and sterilized by three steps of washing orderly with ethanol (95%, *v*/*v*) for 30 s, NaClO (2%, *w*/*v*) for 5 min, and sterile distilled water for eight times. Each sterilized nodule was crushed in a sterilized tube, and the crude extract was streaked onto YMA plates supplied with Tmp and Km. After being fostered for nearly 15 days in a 28°C incubator, isolates were tested by PCR method with two primer pairs 415L/415R (inner primer of Tn*5* transposon) and T5BF/T5BR (external primer of Tn*5* transposon, designed on the base of the *sacB* gene located in downstream of Tn5 transposon) (Table 1). The strains with the positive amplification reaction by primer pair 415L/415R and the negative reaction by T5BF/T5BR were identified as positive mutants. Screened positive mutants were verified using colony purification and nodulation validation for twice or thrice in order to confirm their symbiotic stability on nodulation and nodule numbers with *V. radiata*.

### Mapping and Sequencing Analysis of Transposon Insertion Sites

For identifying the genes mutated by Tn*5* insertion, the transposon insertion sites including the mutated genes were investigated with the following procedure. Total DNA for each Tn*5*-transposon-inserted mutant was extracted using Promega Wizard Genome DNA Purification Kit (Promega, Madison, WI, United States) and digested with *Eco*RI. The digested DNA fragments were precipitated using nucleic acid precipitation kit (Dr. Gen TLE precipitation carrier) from TaKaRa (Dalian, China), then self-ligated by T4 ligase (NEB) and transferred into DH5α competent cells by heat shock. Positive clones with resistance to Km were verified by PCR with inner primers 415L/415R for Tn*5* transposon, and the transposon gene junction region was amplified and sequenced using the specific primer PM (Table 1). To characterize the acquired genes, gene sequences were searched with BLASTX programs at the GenBank database of National Center for Biotechnology Information (NCBI, Bethesda, MD, United States^1^).

### Knockout of Tn*5*-Transposon-Inserted Genes With pJQ200SK Plasmid

In order to exclude false positive of compatible nodulation resulted by the polarity effect derived from Tn*5* transposon insertion mutation, knockout of Tn*5*-transposon-inserted genes were conducted with the triparental conjugation method mentioned above (Liu et al., 2018b), with some modifications, in which the plasmid pRL1063a-2 was replaced with the reformed suicide plasmid pJQ200SK (donor) with the ability of homologous double-crossover recombination. For example, in order to knockout Tn*5*-inserted L82 gene of CCBAU 53363, pJQ200SK-L82 was constructed using the described methods (Quandt and Hynes, 1993; Sha et al., 2001). First, L82 gene with its upstream and downstream sequences were searched and acquired from the complete genome database of CCBAU 53363 using BioEdit and IGV 2.3, respectively (Li et al., 2015). Based on the obtained gene sequences, the two primer pairs L82-1F/L82-1R and L82-2F/L82-2R (Supplementary Table S1) were designed and used to amplify the upstream and downstream DNA fragments of L82 gene, respectively, by PCR method. Second, the two fragments were connected to the *Sma*I restriction site of the suicide plasmid pJQ200SK by seamless cloning, and then, the constructed pJQ200SK-L82 was transformed into DH5α-competent cells of *E. coli*. Third, this plasmid was verified by PCR amplification with primer pair M13F/L82-2R (Supplementary Table S1) to ensure that there was no point mutation in the inserted two fragments and then used as donor in the following triparental conjugation experiment.

During the triparental experiment, the constructed plasmid pJQ200SK-L82 (donor) was introduced into CCBAU 53363 (recipient) with the help of pRK2013 (helper). After triparental conjugating for 4 days, single-crossover transconjugants were selected on TY agar plates containing Gen and Tmp and verified by PCR amplification using the detection forward primer and M13R (L82-F/M13R). The succeeded single-crossover isolates were cultured in TY broth containing Tmp with agitation at 180 rpm for 5 days, and subsequently coated on TY agar supplied with Tmp and sucrose for double-crossover filtering. Double-crossover transconjugants were verified by PCR amplification with external (L82SF/L82SR, positive) and intra (L82NF/L82SR, negative) PCR primers. Isolates were purified three times on TY agar with Tmp and sucrose.

### Symbiotic Phenotype Analysis

Symbiotic phenotypes on *V. radiata* were tested by inoculating separately with the wild-type strain CCBAU 53363, acquired Tn*5*-inserted mutants and gene knockout mutants, Type I strain CCBAU 51778 (as positive control), and 0.8% NaCl solution (as negative control). *V. radiata* seeds were dipped 1 min in 95% ethanol solution for surface dehydration and then sterilized in 2.5% (*w*/*v*) NaClO solution for 8 min. After being rinsed in sterile distilled water for eight times, seeds were transferred onto 0.6% agar–water plates and germinated for 2 days at 28°C. Seedlings in Leonard jars were inoculated with 1 ml of rhizobial suspension with the concentration of OD_600_ = 0.2. Plant chlorophyll content, shoot dry weights, nodule numbers, and nodule fresh weights of all treatments were recorded 30 dpi (Jiao et al., 2015), and nitrogenase activity per plant of each treatment was also measured with exception for that of Tn*5*-inserted mutants’ treatments (Liu et al., 2017). Each treatment consisted of 10 plants in triplicate. Data were processed with Duncan’s *t* test (*P* = 0.05) by SPSS.

### Phylogenetic Analysis and Modeling of Proteins

For understanding the mutated genes’ function and phylogenetic correlations, the authorized mutated genes’ protein sequences of CCBAU 53363, homological protein sequences of the closely related strains, and the two representative strains for types I and II were acquired by searching corresponding genes through BLASTX in National Center for Biotechnology information (NCBI) website. Phylogenetic tree, based on each mutant’ protein sequences of the strain CCBAU 53363 and the homological sequence of the closely related strains, was built respectively by maximum likelihood (ML) method in MEGA 5.05 (Tamura et al., 2011), and the identity percentages were calculated by Poisson correction model. In the same way, phylogenetic trees based on each mutated genes and corresponding protein sequences of the strain CCBAU 53363 and the representative strains for types I and II were separately constructed as well. Bootstrap analyses were performed using 1,000 replicates, and only the bootstraps values > 60% were indicated in the corresponding nodes of the trees. Protein 3D models were predicted by SWISS-MODEL web server and Pymol software.

## Results

### Characterization of the Seven Mutants of CCBAU 53363

To investigate molecular mechanisms underlying unstable nodulation of *B. guangxiense* CCBAU 53363 on *V. radiata* plants, a library containing about 4.5 × 10^7^ Tn*5*-transposon-inserted mutants was created. From 400 *V. radiata* plants inoculated with Tn*5* transposon mutant library, 647 Tn*5*-transposon-inserted mutants of CCBAU 53363 presented increase in nodule numbers comparing with that of the wild-type strain CCBAU 53363, and they were preliminary isolated and purified. Then, 53 out of the 647 mutants were verified to have a better nodulation capability than the others through reinoculation to this plant twice three times, since they showed stable compatibility with *V. radiata*. The knockout mutants of the 53 genes were further constructed through triparental conjugation method, and 7 of the 53 genes were ultimately demonstrated to be responsible for the incompatible symbiosis with *V. radiata* by nodulation tests. By mapping and sequence analysis of the seven mutants of CCBAU 53363, the characteristics including seven mutation gene length and product, protein accession number, and amino acid sequence identities (%) with that of the closed related strains are shown in Table 2. The mutants L265 and L615 were tentatively considered as acyltransferase and hypothetical protein due to their low amino acid sequence identity of 27–35.9% and 10.4–27.8%, respectively with the known proteins of some strains of *Bradyrhizobium* spp. and *Phenylobacterium zucineum*. Another two protein products derived from mutated genes L4 and L82 shared 88.5–92.1% and 77.9–84.2% amino acid identities with some hypothetical proteins of *Bradyrhizobium* spp. Mutant L147, Tn*5* insertion in the 1,113-bp open reading frame (ORF) encoding alanine dehydrogenase, shared the highest identity of 91.5% with AlaDH protein sequence of *Bradyrhizobium* sp. WSM4349 (WP_018459455.1). A product of gene L373, a Tn*5* insertion in the 978-bp ORF encoding tripartite tricarboxylate transporter substrate binding protein (TTT SBP), had the greatest identity of 96.1% with the TTT SBP protein sequence of *Bradyrhizobium* sp. BK707 (WP_130362841.1). The predicted protein of L646 mutant, a Tn*5* insertion in the 867-bp ORF encoding sulfur oxidation c-type cytochrome SoxA, shared 96.1% amino acid sequence identity with SoxA of *B. zhanjiangense* CCBAU 51787 (WP_164934866.1).

**TABLE 2:** Characteristics of the seven mutated genes in the study.

**Gene**	**Length (bp)**	**Annotation**	**Protein accession no.**	**Strain (protein accession no., identity percentage^a^)**
L4	1,305	Hypothetical protein	WP_164937829.1	*B. nanningense* CCBAU 51757 (WP_164936447.1, 92.1%) *Bradyrhizobium* sp. INPA54B (WP_100231454.1, 90.8%) *Bradyrhizobium* sp. WSM1743 (WP_156952278.1, 90.1%) *B. shewense* ERR11 (WP_165637841.1, 88.5%) *Bradyrhizobium* sp. TSA1 (PIT04988.1, 88.5%)
L82	291	Hypothetical protein	WP_128925684.1	*Bradyrhizobium* sp. MOS003 (WP_106950693.1, 84.2%) *Bradyrhizobium* sp. AC87j1 (WP_104462656.1, 83.0%) *Bradyrhizobium* sp. Rc3b (WP_092258140.1, 83.0%) *Bradyrhizobium* sp. WSM2793 (WP_018320481.1, 81.8%) *Bradyrhizobium* sp. TSA1 (WP_100174730.1, 77.9%)
L147	1113	Alanine dehydrogenase	WP_128929853.1 (*ald*)	*Bradyrhizobium* sp. WSM4349 (WP_018459455.1, 91.5%) *Bradyrhizobium* sp. DOA9 (WP_025038509.1, 91.2%) *Bradyrhizobium* sp. aSej3 (WP_148742750.1, 91.0%) *Bradyrhizobium* sp. LMTR 3 (WP_065746120.1, 91.0%) *Bradyrhizobium sacchari* BR10555 (WP_080137720.1, 89.2%)
L265	1113	Acyltransferase	WP_128928564.1	*Bradyrhizobium* sp. 63S1MB (QIO34022.1, 35.9%) *Bradyrhizobium* sp. BK707 (WP_130363856.1, 29.8%) *Bradyrhizobium* sp. cf659 (WP_092190185.1, 27.0%)
L373	978	Tripartite tricarboxylate transporter substrate binding protein	WP_128928985.1	*Bradyrhizobium* sp. BK707 (WP_130362841.1, 96.1%) *Bradyrhizobium sacchari* BR10555 (WP_080137426.1, 95.0%) *Bradyrhizobium* sp. WSM2254 (WP_084302701.1, 92.3%) *Bradyrhizobium* sp. MOS001 (WP_135216717.1, 91.7%) *Bradyrhizobium* sp. CNPSo 3448 (WP_135178836.1, 91.7%)
L615	852	Hypothetical protein	WP_128923995.1	*Phenylobacterium zucineum* (PZQ60673.1, 27.8%) *B. lablabi* MT34 (SHK62758.1, 10.4%)
L646	867	Sulfur oxidation c-type cytochrome SoxA	WP_164938020.1 (*soxA*)	*B. zhanjiangense* CCBAU 51787 (WP_164934866.1, 96.1%) *Bradyrhizobium* sp. Rc3b (SFM39897.1, 95.7%) *Bradyrhizobium* sp. Rc3b (WP_092251643.1, 95.7%) *Bradyrhizobium* sp. WSM2254 (WP_084301459.1, 94.3%) *B. vignae* LMG 28791 (WP_122406152.1, 92.0%)

*^*a*^Identity percentages were obtained by the phylogenetic analysis of Mega 5.05 ML Analysis.*

### Symbiotic Phenotypes of Tn*5*-Transposon-Inserted Mutants on *V. radiata* Plant

In symbiotic test, the symbiosis between Type I strain CCBAU 51778 (positive control) and *V. radiata* was stable or effective, which formed deep red interior nodules and dark green leaves, and the plants showed that chlorophyll content, nodule numbers, and fresh weight and shoot dry weight were significantly higher than those of the other plants. On the other hand, wild-type strain CCBAU 53363 showed incompatible symbiosis, as expressed by the following: (1) no nodules appeared in ∼40% of the inoculated plants, and the other 60% plants formed one to three pink nodules, which evidenced the incompatible nodulation and (2) it showed significantly lower chlorophyll content and shoot dry weight than that of CCBAU 51778, which were similar to that of the non-inoculated controls. Significantly, the seven Tn*5*-transposon-inserted mutants increased nodule number and nodule fresh weight on the inoculated plants, indicating the stable or effective nodulation capacity when compared with the wild-type CCBAU 53363, but still a little bit lower than that of the Type I strain CCBAU 51778, except of the L373-T mutant. Generally, the rhizobial gene mutations did not influence plant chlorophyll content and shoot dry weight compared with the wild-type strain. Therefore, these mutated genes were preliminarily speculated to participate in negatively regulating nodulation of CCBAU 53363 with *V. radiata* (Supplementary Figures S1, S2).

### Symbiotic Phenotypes of Gene Knockout Mutants on *V. radiata* Plant

**FIGURE 1:**
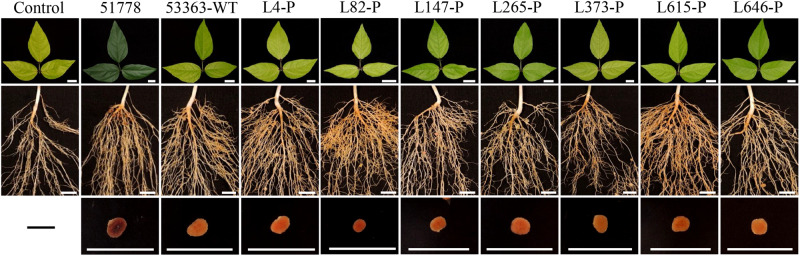
Leaves, roots, and nodules of *Vigna radiata* inoculated with Type I strain *B. guangdongense* CCBAU 51778 (51778), Type II wild-type strain *B. guangxiense* CCBAU 53363 (53363-WT), and gene knockout mutants of CCBAU 53363 with the help of pJQ200SK (gene number-P). Each treatment consisted of 10 plants in triplicate. Plants were harvested at 30 dpi. Scar bars: 1 cm.

**FIGURE 2:**
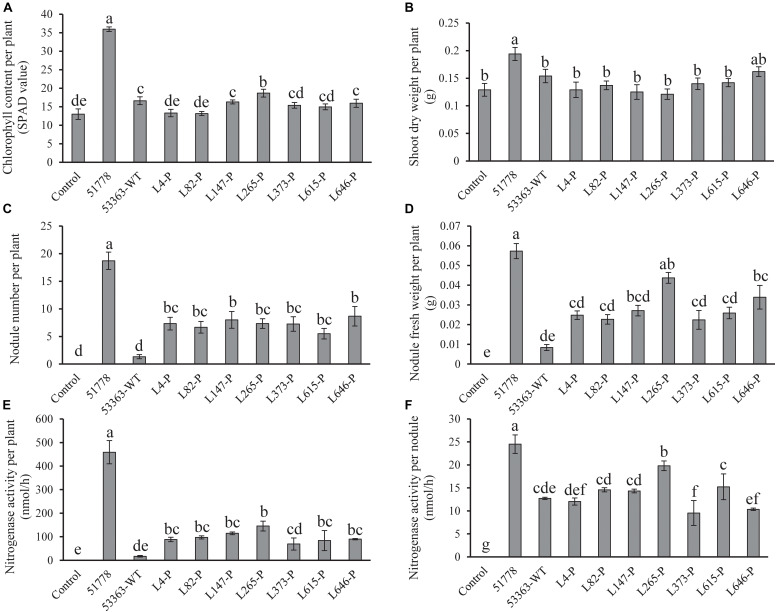
Symbiotic properties of *V. radiata* without inoculation (control) and inoculated with Type I strain *B. guangdongense* CCBAU 51778 (51778), Type II wild-type strain *B. guangxiense* CCBAU 53363 (53363-WT), and gene knockout mutants of CCBAU 53363 with the help of pJQ200SK (gene number-P). **(A)** Plant chlorophyll content, **(B)** shoot dry weight, **(C)** nodule number, **(D)** nodule fresh weight, **(E)** nitrogenase activity per plant, and **(F)** nitrogenase activity per nodule were measured at 30 dpi. Each treatment consisted of 10 plants in triplicate, and values were means of 10 plants. Different letters indicated significant difference based on Duncan’s *t* test (*P* = 0.05).

Seven mutated genes mentioned above were completely knockout by plasmid pJQ200SK, and symbiotic phenotype verification was performed with newly constructed mutants separately inoculated on *V. radiata*. Results showed that, with the exception of L373-P, symbiotic phenotypes of *V. radiata* inoculated with the other six gene knockout mutants (L4-P, L82-P, L147-P, L265-P, L615-P, L646-P) were the same as that of their Tn*5*-transposon-inserted mutants, demonstrating that they were responsible for the stable or effective nodulation of CCBAU 53363 with *V. radiata*. Nodule number and nodule fresh weight of *V. radiata* induced by L373-P mutant were remarkably lower than that of L373-T mutant but still more than that of wild-type strain CCBAU 53363, suggesting that L373-T mutant resulted in a polar effect to some extent but the knockout mutant L373-P were verified to negatively regulate nodulation of CCBAU 53363 with *V. radiata*. The result confirmed the association of the seven mutated genes with nodulation incompatibility on *V. radiata*; however, comparing with CCBUA 53363, the increased nodule number and nodule fresh weight induced by the seven mutants had no significant effects on plant chlorophyll content and shoot dry weight, implying that the problem of plant nitrogen deficiency had not been thoroughly solved (Figures 1, 2A–D and Supplementary Figure S2).

In order to determine relations between mutated genes and the nitrogen fixation efficiency of nodules, as well as the increased nodule number and plant nitrogen deficiency phenotypes, we tested nitrogenase activity of nodules induced by CCBAU 51778, CCBAU 53363, and mutant inoculated plants, respectively. The results (Figure 2E) showed that nitrogenase activity per plant inoculated with mutants were significantly higher than that of the wild-type CCBAU 53363, with exception of mutant L373-P, but lower than that of Type I strain CCBAU 517787. It might explain that nitrogen fixed by the mutant-induced nodules could not completely meet the necessity for plant growth. As to nitrogenase activity per nodule (Figure 2F), CCBAU 53363 and its seven mutants showed significantly lower level of activity than CCBAU 51778, and the four mutants L4-P, L82-P, L147-P, and L615-P presented similar nitrogen-fixing capacity with the original strain CCBAU 53363, implying that the four mutants were not associated with nitrogen-fixing efficiency. L265-P showed significantly higher nitrogenase activity, whereas L373-P was lower than CCBAU 53363, implying that L265-P or L373-P might have positive or negative correlations with nitrogen-fixation efficiency.

### Nucleotide Sequence Analysis of Mutation Genes of Type I and II Strains

The seven symbiotic-related genes detected in CCBAU 53363 were identified to have negative regulatory effects on its nodulation with *V. radiata* in this study. Furthermore, we collected and aligned these gene sequences of CCBAU 53363 with those of the other Type I and II strains to find the differences separately shared by the strains in each type, which might be the foremost reason for incompatible symbiosis of CCBAU 53363 and the other Type II strains with *V. radiata*. For this analysis, genomes of the Type I strains CCBAU 51757, CCBAU 51778, CCBAU 51787, and CCBAU 53390, and the Type II strains CCBAU 51649 and CCBAU 51670 were used. Results (Supplementary Table S2) displayed that L4, L82, L373, and L646 genes were located in the chromosomes of all the tested strains with one copy, except for CCBAU 51649 in which the L373 gene was missing, while L265 and L615 were recognized as unique genes of CCBAU 53363 with the ability of causing restrictive nodulation on *V. radiata* plant. Through sequence comparison in this study, we found that Type II strains possessed two copies of L147 gene, which were located in the symbiotic plasmid (L147-p, identical to that of the inserted/knockout gene of CCBAU 53363) and chromosomal symbiotic gene cluster (L147-c, 66.3–66.9% identity with L147-p of CCBAU 53363), respectively. However, only one copy of its homolog (L147-c) was identified in the chromosomal symbiotic gene cluster of type I strains.

Further phylogenetic analyses based on the nucleotide sequences of these genes were performed to verify the evolutionary correlations between Type I and II strains. Results showed that genes L4, L82, L373, and L646 of CCBAU 53363 shared high level identities of 81.8–91.4%, 88.3–94.8%, 81.9–83.4%, and 87.7–92.7% (≥ 80%) with homologous genes of the other tested strains (Supplementary Table S2). L147-p gene of CCBAU 53363 was identical to that in the symbiotic plasmid of Type II strains CCBAU 51649 and CCBAU 51670 and was more different from that (70.7–74.1% identity) in chromosomes of Type I strains and L147-c (66.8–69.3% identity) of Type II strains (Supplementary Table S2). Within strains in each of the Type I and II or between the Type I and II strains, L373 and L646 genes shared sequence identities of 82.8–100%, 81.9%, and 81.4–83.4% for L373 (Supplementary Table S3) and 92–100%, 87.7–91.6%, and 88.2–92.7% for L646 (Table 3), respectively, indicating that there was no major difference (identities ≥ 81.8%) of the chromosome genes within or between the Type I and II strains. Results for L4 and L82 were not shown due to similar properties of high-level gene nucleotide identities (≥ 81.8%) and one copy gene in the chromosome among two types of strains compared with that of the genes L373 and L646. In the same analysis for L147-c or L147-p of the Type II strains and L147-c of the Type I strains, the situation was complicated to some extent (Table 4). L147-p gene in the symbiosis plasmid of CCBAU 53363 was identical to that in the symbiosis plasmid of Type II strains CCBAU 51649 and CCBAU 51670 and was different from L147-c in the chromosomes of Type I and II strains with identities of 70.7–74.1% and 66.8–69.3%, respectively. Sequence identities were 75–93.1% for chromosome gene L147-c among the Type I strains, 85.7–86.4% for L147-c among the Type II strains, and 62.5–67.2% for L147-c between the Type I and II strains. Interestingly, L147-p was lightly closely related to L147-c in the chromosome of Type I than L147-c of Type II strains, implying that the two copies of L147 gene in Type II strains might have different evolutionary histories. In general, the mutated genes in the chromosome between Type I and II strains had minor differences (identities ≥ 81.8%) compared with that between the homologous genes in the Type II unique symbiotic plasmid and in the chromosome (identities ≤ 74.1%) of both Type I and II strains.

**TABLE 3:** Identity percentages (%) of the L646 gene sequence among the tested *Bradyrhizobium* strains.

**L646 gene**	**Type I**				**Type II**		
	**51757^a^**	**51778**	**51787**	**53390**	**51649**	**51670**	**53363**
**Type I**							
51757	100.0	92.0	92.0	100.0	90.2	88.2	92.7
51778	92.0	100.0	100.0	92.0	91.2	89.1	91.9
51787	92.0	100.0	100.0	92.0	91.2	89.1	91.9
53390	100.0	92.0	92.0	100.0	90.2	88.2	92.7
**Type II**							
51649	90.2	91.2	91.2	90.2	100.0	91.6	90.2
51670	88.2	89.1	89.1	88.2	91.6	100.0	87.7
53363	92.7	91.9	91.9	92.7	90.2	87.7	100.0

*^*a*^CCBAU strain number. Identities (%) were calculated by using MEGA 5.05.*

**TABLE 4:** Identity percentages (%) of the L147 gene sequences among the tested *Bradyrhizobium* strains.

**L147 gene**	**Type I**				**Type II**			
	**51757-c^a^**	**51778-c**	**51787-c**	**53390-c**	**51649-c**	**51670-c**	**53363-c**	**53363-p**
**Type I**								
51757-c	100.0	89.8	75.0	90.4	62.5	63.4	64.5	70.7
51778-c	89.8	100.0	78.5	93.1	65.9	66.5	67.2	74.1
51787-c	75.0	78.5	100.0	76.0	65.5	65.7	66.3	73.8
53390-c	90.4	93.1	76.0	100.0	63.2	64.9	66.6	73.5
**Type II**								
51649-c	62.5	65.9	65.5	63.2	100.0	85.8	85.7	66.9
51670-c	63.4	66.5	65.7	64.9	85.8	100.0	86.4	66.8
53363-c	64.5	67.2	66.3	66.6	85.7	86.4	100.0	69.3
^b^ 53363-p	70.7	74.1	73.8	73.5	66.9	66.8	69.3	100.0

*^*a*^All strains number are CCBAU strain number. Strain number with c indicates the gene L147 locating in chromosome, with p locating in symbiotic plasmid. Due to similarities of L147 gene were total identical in Type II strains symbiotic plasmid, we only used CCBAU 53363-p as representative strain of Type II strains symbiotic plasmid. Identities (%) were calculated using MEGA 5.05.*

### Phylogenetic Tree and 3D Structure Prediction of Symbiotic-Related Proteins

**FIGURE 3:**
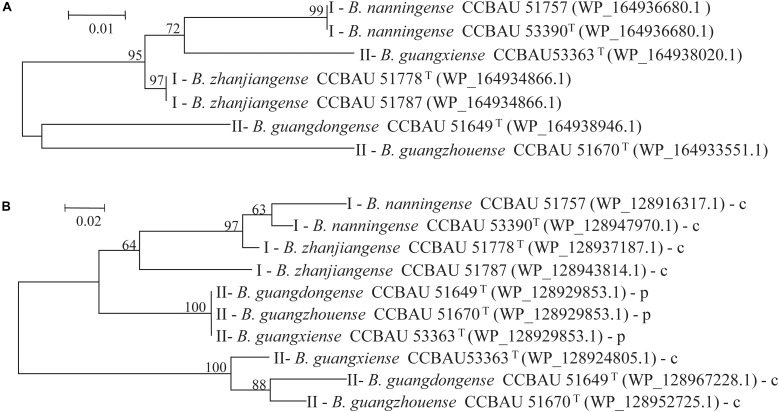
Phylogenetic trees based on amino acid sequences of proteins **(A)** L646 and **(B)** L147 of two type representative strains were constructed by maximum likelihood (ML) method using MEGA 5.05. Bootstrap values > 60% from 1,000 replicates are indicated at branches. GenBank accession numbers are within brackets. **(B)** p or c next to strain numbers represent L147 genes location in plasmid or chromosome.

**FIGURE 4:**
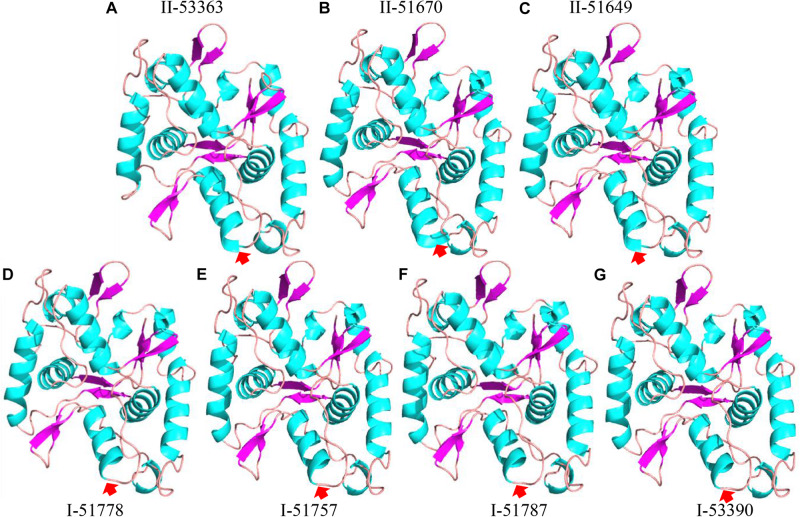
Protein 3D structure predictions for L646 gene in Type II strains **(A)** CCBAU 53363, **(B)** CCBAU 51670, and **(C)** CCBAU 51649; and Type I strains **(D)** CCBAU 51778, **(E)** CCBAU 51757, **(F)** CCBAU 51787, and **(G)** CCBAU 53390 using SWISS-MODEL server and Pymol software.

To further analyze the effects of genetic differences between Type I and II strains on protein phylogenetic relationships, 3D structures, and functions, we performed amino acid sequence alignments and constructed phylogenetic trees, as well as predicted 3D structures for L147, L373, and L646 proteins. It was shown that the phylogenies for L373 and L646 amino acid sequences were very similar, and all strains were divided into two branches: one consisted of the Type II strain CCBAU 53363 and all the Type I strains with the identities of 87.9–100% for L373 proteins and 94.6–100% for L646 proteins; another one included the remaining Type II strains CCBAU 51670 and CCBAU 51649 (only for L646) with identities of 92.4% for L646 proteins (Figure 3A, Supplementary Figure S3, and Supplementary Tables S4, S5). However, protein 3D structures in the two types were not significantly affected, and only minor difference in one α-helix (red arrow) was found (Figure 4 and Supplementary Figure S4). These results indicated that the differences in amino acid sequences deduced from the mentioned chromosome genes in the Type I and II strains had no great effects on the 3D structure of the proteins.

**FIGURE 5:**
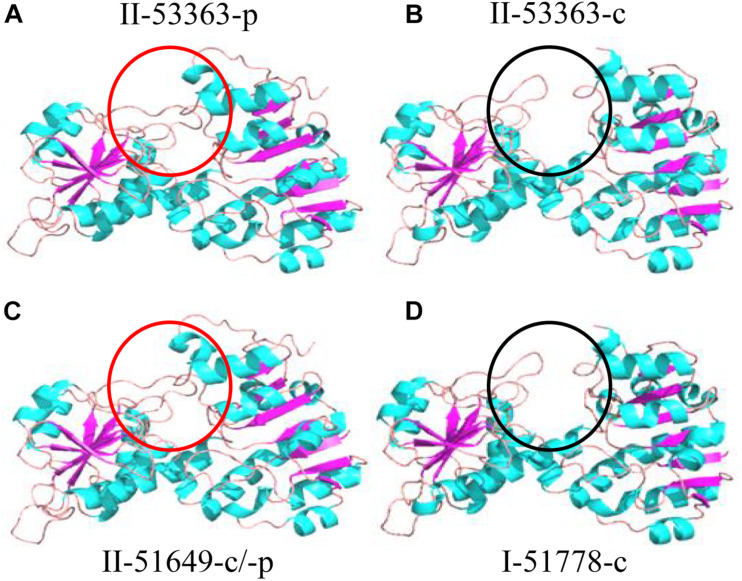
Protein subunit 3D structure predictions for L147 gene by the representatives of Type II strains **(A)** CCBAU 53363 plasmid, **(B)** CCBAU 53363 chromosome, **(C)** CCBAU 51649 chromosome/plasmid, and **(D)** Type I strain CCBAU 51778 chromosome using SWISS-MODEL server and Pymol software. The part covered by a circle indicates different sites among strains. Same color circles present the same 3D structure at the site.

Phylogeny of amino acid sequences deduced from the L147 genes classified the tested strains into three clades represented respectively by the Type I strains, symbiotic plasmid copy of the Type II strains, and chromosome symbiotic gene copy of the Type II strains, with inner-clade identities of 87.6–96.7%, 100%, and 93.3–94.7% amino acid sequences (Figure 3B and Supplementary Table S6). However, analysis of protein 3D structures (subunit and hexamer) identified them into two categories: category 1 covered the Type II strains CCBAU 53363-p (gene in plasmid), CCBAU 51649-c (gene in chromosome) and CCBAU 51649-p (the same structure), and CCBAU 51670-c and CCBAU 51670-p (the same structure); category 2 consisted of all the Type I representative strains (CCBAU 51778-c, CCBAU 51757-c, CCBAU 51787-c, CCBAU 53390-c) and the Type II strain CCBAU 53363-c (Figure 5 and Supplementary Figures S5, S6). These results indicated that protein 3D structures were not identical with their amino acid sequences phylogeny, which might be caused by amino acid residues, polarities, and hydrophobicity, which affected the folding of amino acid sequence into the 3D structure. The main distinctions between the two categories of 3D subunit structures were located on the spatial conformation of catalytic binding groove (red circle for category one and black circle for category two) (Figure 5 and Supplementary Figure S5). Similar to the subunits, the 3D structures of the L147 hexamers also indicated two different spatial structures (red or black rectangle gave an indication of a different site) (Supplementary Figure S6).

In addition, CCBAU 53363 simultaneously possessed both categories of L147 proteins, representing that it synthesized both the chromosome (Type I strain, L147-c) and symbiotic plasmid (Type II strain, L147-p) encoded proteins. However, the other Type II strains only translated L147-p protein. Considering the same symbiotic phenotypes of CCBAU 53363 as Type II strains on *V. radiata* (Li, 2019), it could be concluded that, for strain CCBAU 53363, L147-p protein functioned greater than that of L147-c, meaning that L147 in plasmid played a critical role in negatively regulating the symbiotic compatibility on *V. radiata.*

## Discussion

Contrary to conventional cognition, a previous study demonstrated that Type II peanut bradyrhizobia strains possessed incompatible symbiotic phenotypes with *V. radiata*, a plant belonging to the *A. hypogaea* cross-nodulation group (Li, 2019). Due to the differences between the genomes of strains in types I and II (Li, 2019), reasons for the incompatibility of Type II strains with *V. radiata* plants appear to be a genetic barrier. It is that the inactivation of genes associated with the rhizobial negative factor allowed the mutants to overcome the nodulation restriction conferred by plant and successfully achieve symbiosis, similar to the incompatible symbiosis between soybean plants carrying Rj4 and USDA 61 strain (Faruque et al., 2015). This study used Tn*5* mutagenesis to screen for mutants of Type II strain CCBAU 53363 compatible with *V. radiata* to investigate the genetic mechanisms of its incompatibility with *V. radiata*. Successful isolation of seven mutants with the ability of stable nodulation with *V. radiata* (Figures 1, 2 and Supplementary Figures S1, S2), comparative analysis results of the mutation genes’ sequence (L373, L646, and L147) (Tables 3, 4 and Supplementary Tables S2, S3) and corresponding amino acid sequences (Supplementary Tables S4–S6), amino acid sequence phylogenetic trees (Figure 3 and Supplementary Figure S3), and protein 3D structure predictions (Figures 4, 5 and Supplementary Figures S4, S5) in the present study initially supported our speculation that genetic barrier, caused by the presence of seven genes in the Type II strains, is a crucial cause of the incompatible symbiosis. However, generally, mutants’ lower efficient nitrogen fixation ability in nodules showed that other barriers also play roles in the incompatible symbiosis between the Type II strains and *V. radiata*, which needed to be further studied.

The mutation of L147 was located in the gene of encoding L-alanine dehydrogenase (AlaDH, EC 1.4.1.1) that participates in producing alanine from pyruvate and NH_3_/NH_4_^+^ in *B. japonicum* strain 110 bacteroids inside the soybean nodules, but it is not essential for symbiosis (Lodwig et al., 2004). This enzyme influences amino acid cycle and pyruvate metabolism level in *Rhizobium leguminosarum* cells through the alanine synthesis, which in turn affects the plant nitrogen content and the rhizobial system of tricarboxylic acid cycle and ultimately affects the plant biomass accumulation in soybean plant and bacteroid metabolism in pea nodules (Smith and Emerich, 1993; Lodwig et al., 2004; Dave and Kadeppagari, 2019). Thus, we can conclude that AlaDH participates in the complex metabolic regulation network of rhizobia. However, it remains unclear how AlaDH regulates nodulation or host infection of rhizobia. In this research, comparison analysis demonstrated that L147-p functioned stronger than L147-c in CCBAU 53363. Symbiotic test found that L147-p gene knockout mutant of CCBAU 53363 did not affect chlorophyll content, shoot dry weight, and nodule’s nitrogenase activity, indicating that both L147-p and L147-c did not promote plant biomass accumulation or enhance nodule N_2_-fixation efficiency in the case of plant nitrogen deficiency. However, this mutant enhanced nodule number and nodule fresh weight, illustrating that L147-p negatively regulated rhizobial nodulation with *V. radiata*. The role of AlaDH in regulating rhizobial nodulation has not been reported before; therefore, we first found that AlaDH plays a significant role in regulating rhizobial stable nodulation with legume under the premise of plant nitrogen deficiency, which may be affected by the cell regulatory network, and detailed mechanisms needed to be further detected. Furthermore, the mutation of L147-c or double mutation of L147-c-p gene of CCBAU 53363 will help us better understand the function of L147 gene and its regulation mechanism.

The L373 mutant was located in the gene of translating TTT SBP. TTT family is a poorly characterized group of prokaryotic secondary solute transport systems, which employ a periplasmic SBP for initial ligand recognition and present in many bacteria (Winnen et al., 2003; Rosa et al., 2017). SBPs bind with high affinity to diverse classes of substrates, such as tricarboxylates, amino acids, nicotinic acid, nicotinamide, and benzoate (Herrou et al., 2007); terephthalate and other aromatics (Hosaka et al., 2013); 3-sulfolactate (Denger and Cook, 2010); and C_4_-dicarboxylic acids (plant secretions, such as succinate, fumarate, and malate, etc.) (Rosa et al., 2019), which deliver substrates to the trans-membrane domains to be imported into the cell. C_4_-Dicarboxylic acids have been shown to play important roles as substrates and signal compounds for rhizobia and are considered to be the major carbon sources utilized by free-living *Rhizobium* species during the colonization on root surface (Robinson and Bauer, 1993). However, there is no research on the relationship between TTT SBP and rhizobial symbiosis with legumes. In this study, with the knockout of L373 gene, mutant significantly stabilized the plant nodulation by enhancing nodule number and nodule fresh weight, but it had no influences on chlorophyll content and shoot dry weight, with decreased nodule nitrogenase activity, when compared with the wild-type strain CCBAU 53363. We speculated that the substrate recognition and absorption system involved in the TTT SBP protein might have no preference for *V. radiata* plant root secretion substrates. With the mutation of TTT SBP protein, rhizobial affinity substrate recognition and absorption system to the plant root secretions were enhanced by some unknown reasons and followed with affected rhizobial colonization and nodulation efficiency on root surface. It is the first finding that TTT SBP substrate absorption system of CCBAU 53363 is involved in regulating compatible symbiosis with *V. radiata* and nodule nitrogen fixation efficiency.

In the mutant L646, a gene encoding sulfur oxidation c-type cytochrome SoxA protein was found to be mutated, which was a subunit of SoxAX cytochromes, a part of c-type cytochromes that catalyzes the transformation of inorganic sulfur compounds (Bamford et al., 2002; Ogawa et al., 2008). Inorganic sulfur compounds’ oxidation capability is frequently found in phylogenetically and physiologically diverse bacteria, including the members of *Bradyrhizobiaceae* with *sox* homologs of *B. japonicum* USDA110 (Masuda et al., 2010). However, the relationship between inorganic sulfur metabolism and symbiosis of rhizobia with plants has not been studied. In this research, *soxA* knockout mutant significantly increased the nodule number and nodule fresh weight, but *V. radiata* plant’s chlorophyll content, shoot dry weight, and nodule nitrogen fixation efficiency were not influenced, indicating that *soxA* negatively regulated CCBAU 53363 nodulation on *V. radiata*, and had no significant effects on the extremely inefficient nitrogen fixation ability. Therefore, a certain regulatory relationship might exist between the inorganic sulfur metabolism of rhizobia and their stable nodulation with legumes.

Studies have demonstrated that a successful symbiosis depends on the interaction of rhizobia and plant with complex chemical signaling communication (Limpens et al., 2003; Madsen et al., 2003; Radutoiu et al., 2003; Arrighi et al., 2006), recruitment and attachment of rhizobia to growing root hair tips (Murray, 2011), activation and inhibition of plant defense systems during the rhizobial infection (Bright and Bulgheresi, 2010; Tóth and Stacey, 2015), and rhizobial differentiation and bacteroids metabolization in plant cells (Oldroyd and Downie, 2008). In this research, the Type II strains occasionally nodulated with *V. radiata*, illustrating the incompatible and unsuccessful interactions between these strains and *V. radiata*. According to the results of mutants’ symbiosis with *V. radiata*, we found that factors with multiple functions in Type II strains participated in the incompatible symbiosis with *V. radiata*, such as substrate recognition and absorption before infection (L373), inorganic sulfur metabolism (L646), and proteins with unknown functions (L4, L82, L265, L615). At the same time, with the exception of new genes, we also discovered new functions for some well-known genes. For example, during the incompatible symbiosis of CCBAU 53363 and *V. radiata* (in the case of plant nitrogen deficiency), L147 gene did not function as mentioned in other studies (Lodwig et al., 2004) but participated in the negative regulation of stable nodulation. Our mutant screening analysis identified several genetic factors of CCBAU 53363 involved in the incompatibility with *V. radiata* and implied that diverse and multiple mechanisms might cause these host-specific interactions.

Further protein prediction revealed that the 3D structure (category 1) deduced by symbiosis-related gene L147-p of CCBAU 53363 and L147-c/-p of other Type II strains was different from that of the corresponding protein (category 2) coded by chromosome genes in Type I and II strains. The difference might be the crucial site affecting L147 protease catalytic activity or efficiency and would be directly related to the symbiotic phenotype divergence between Type II and Type I strains. It could be supposed that the gene L147-p in CCBAU 53363 functioned stronger than the L147-c counterpart, although it needs to be further confirmed through experiments. According to L373 and L646 genes and the other symbiosis-related genes, the chromosome genes of Type II strains possessed high genetic homology and similar 3D protein structures with the corresponding genes in chromosome of Type I strains, which illustrated that they might indirectly regulate the symbiosis by an unknown way and eventually led to this incompatible symbiosis on *V. radiata*. It is therefore likely that the genetic barrier exists between Type II strains and *V. radiata*: the mutated genes associated with rhizobial negative factor directly or indirectly allowed mutants to overcome the condition of unstable nodulation, a part of incompatible symbiotic barrier.

In brief, the present study initially demonstrated seven genes of Type II strain CCBAU 53363 responsible for compatible nodulation with *V. radiata*, in which the regulation mechanism is needed to be further researched. These results partially explained the restrictive nodulation of CCBAU 53363 with *V. radiata*. Simultaneously, we also found that the L265 gene negatively regulated nodule nitrogenase activity. However, L265-T/P mutant inoculated plants were still in nitrogen deficiency state due to the low number of nodules, indicating that trials on enhancing nodules number will be needed. Besides, with the comparison of original strain CCBAU 53363, L4, L82, L147, L615, and L646 gene knockout mutants did not influence the nodule nitrogen fixation efficiency, which implied that the experiments on improving nodule nitrogenase activity need to be implemented. That is, Tn*5* transposon randomly inserted in mutants’ genome and selected by *V. radiata* with the different standards mentioned above will help us to explore the determinants and regulatory networks for incompatible symbiosis between Type II strains and *V. radiata*; Songwattana et al. (2019) demonstrated that the photosynthetic bradyrhizobial strain ORS278 acquired a broader host range with the ability to form nodules on *Crotalaria juncea* and *Macroptilium atropurpureum* through acquiring a symbiotic mega-plasmid from the non-photosynthetic *Bradyrhizobium* strain DOA9. Otherwise, the “experimental evolution” approaches used by Marchetti et al. (2010) evolved a plant pathogen into legume symbiont after transferring a symbiotic plasmid. All these data verified that the symbiotic plasmid played a great role in rhizobial host range and symbiotic compatibility with plants. Coincidentally, we found that gene in plasmid functioned stronger than the copy in chromosome in CCBAU 53363, and only Type II strains contain symbiotic plasmid with identical nucleotide sequences (Li, 2019). Therefore, symbiotic plasmid might also play a role in Type II strains’ symbiotic compatibility with *V. radiata*. It would be an interesting and positive certification for this supposes to transfer the symbiotic plasmid from Type II to Type I strains, as Type I strains obtain plasmid and show incompatible symbiosis similar to type II strains.

## Data Availability Statement

Publicly available datasets were analyzed in this study. This data can be found here: CP022219.1, CP030053.1, CP030051.1, CP022221.1, LBJQ00000000, LBJM00000000, LBJC00000000, WP_128916317.1, WP_128937187.1, WP_128943814.1, WP_12 8947970.1, WP_128967228.1, WP_128952725.1, WP_1289248 05.1, WP_128929853.1, WP_128918822.1, WP_128930686.1, WP_128947785.1, RXH23619.1, WP_128949221.1, WP_1289289 85.1, WP_164936680.1, WP_164934866.1, WP_164938946.1, WP_164933551.1, and WP_164938020.1.

## Author Contributions

YW, XS, and YL conceived the study. YW, JS, LC, and BH performed the experiments. YW and XS analyzed the data and wrote the manuscript along with the help of EW. CT, WFC, and WXC provided resources. All authors contributed to the article and approved the submitted version.

## Conflict of Interest

The authors declare that the research was conducted in the absence of any commercial or financial relationships that could be construed as a potential conflict of interest.

## Footnotes

1 http://blast.ncbi.nlm.nih.gov/Blast.cgi
