# Supplementary material for: Multiple Genes of Symbiotic Plasmid and Chromosome in Type II Peanut Bradyrhizobium Strains Corresponding to the Incompatible Symbiosis With Vigna radiata
Source: Front Microbiol. 2020 Jun 23;11:1175. doi: 10.3389/fmicb.2020.01175 (PMC7324677; doi:10.3389/fmicb.2020.01175)
Supplement: Supplementary file 1 [file Image_1.PDF]

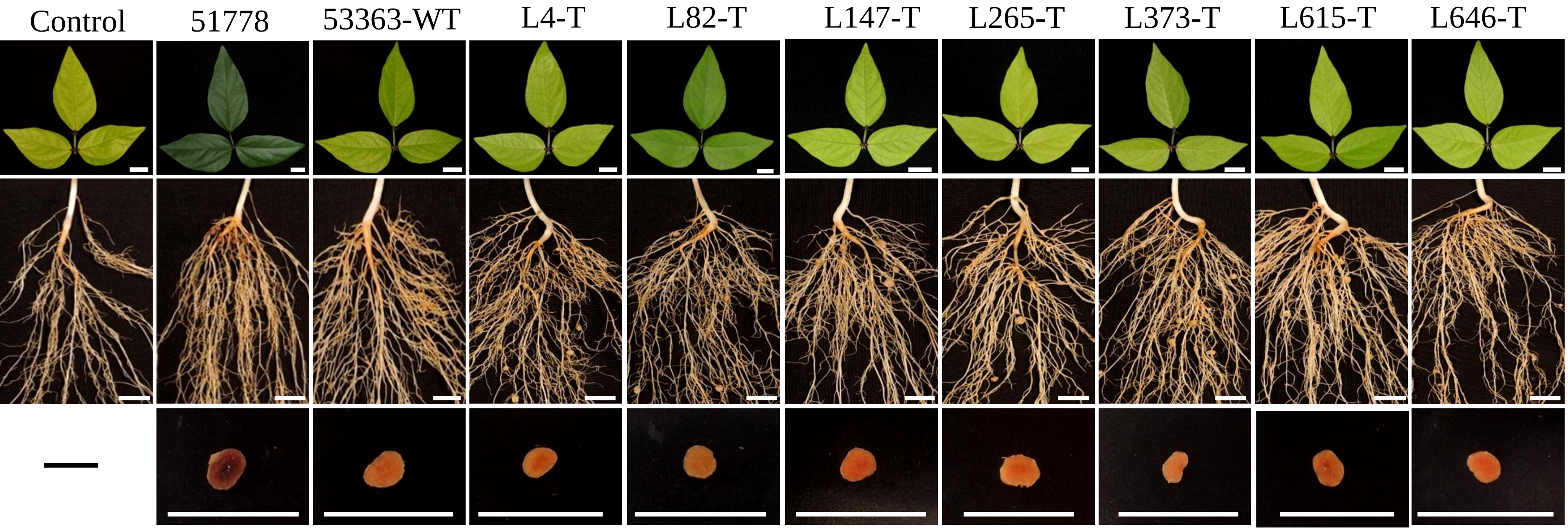

Supplementary Figure S1 Leaves, roots and nodules of *Vigna radiata* inoculated with Type I strain *B. zhanjiangense* CCBAU 51778 (51778), Type II wild-type strain *B. guangxiense* CCBAU 53363 (53363-WT) and CCBAU 53363 Tn*5* inserted mutants (number-T). Plants were harvested at 30 days post inoculation. Scar bars: 1cm.

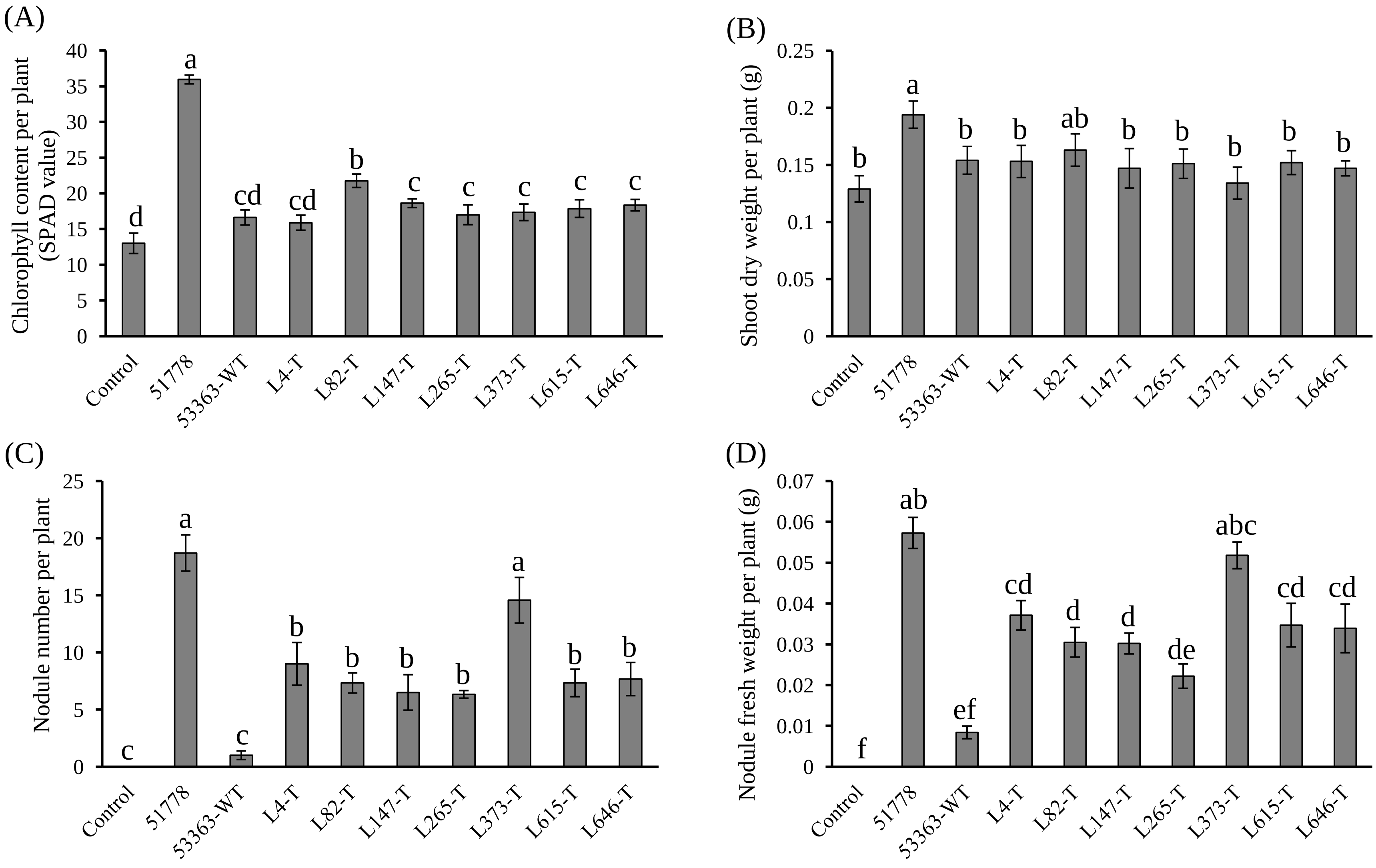

Supplementary Figure S2 Symbiosis properties of *V. radiata* inoculated with Type I strain *B. zhanjiangense* CCBAU 51778 (51778), Type II wild-type strain *B. guangxiense* CCBAU 53363 (53363-WT) and CCBAU 53363 Tn*5* inserted mutants (number-T). Chlorophyll content (A), shoot dry weight (B), nodule number (C) and nodule fresh weight (D) per plant were measured at 30 days post inoculation. The tests were performed three time and values are means of ten plants. Different letters indicated significant difference based on Duncan’s *t* test (*P*=0.05).

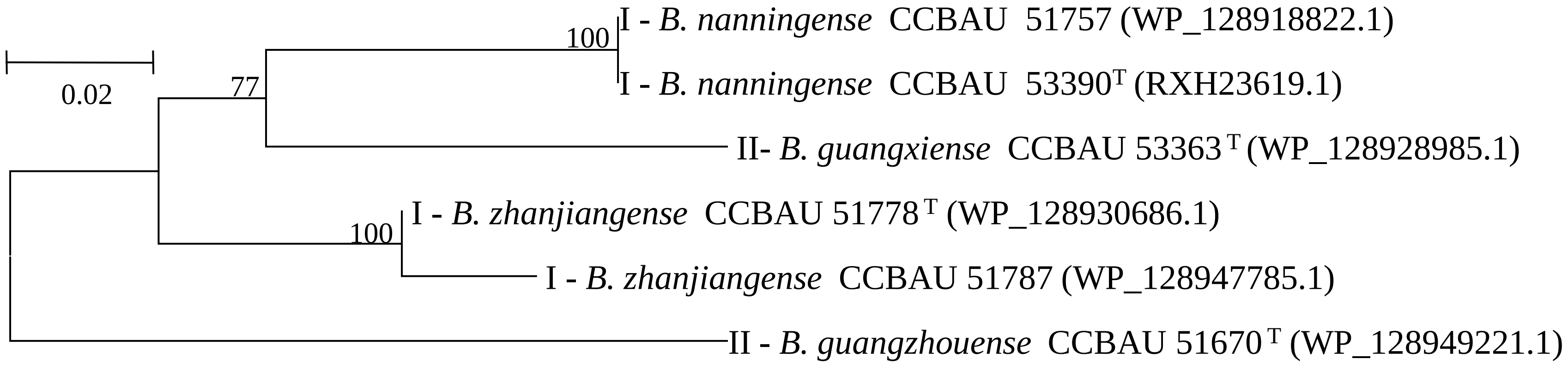

Supplementary Figure S3 Phylogenetic tree based on amino acid sequences of L373 proteins of two type representative strains was constructed by ML method using MEGA 5.05. Bootstrap values greater than 60% from 1000 replicates were indicated at branches. Protein accession numbers are within brackets.

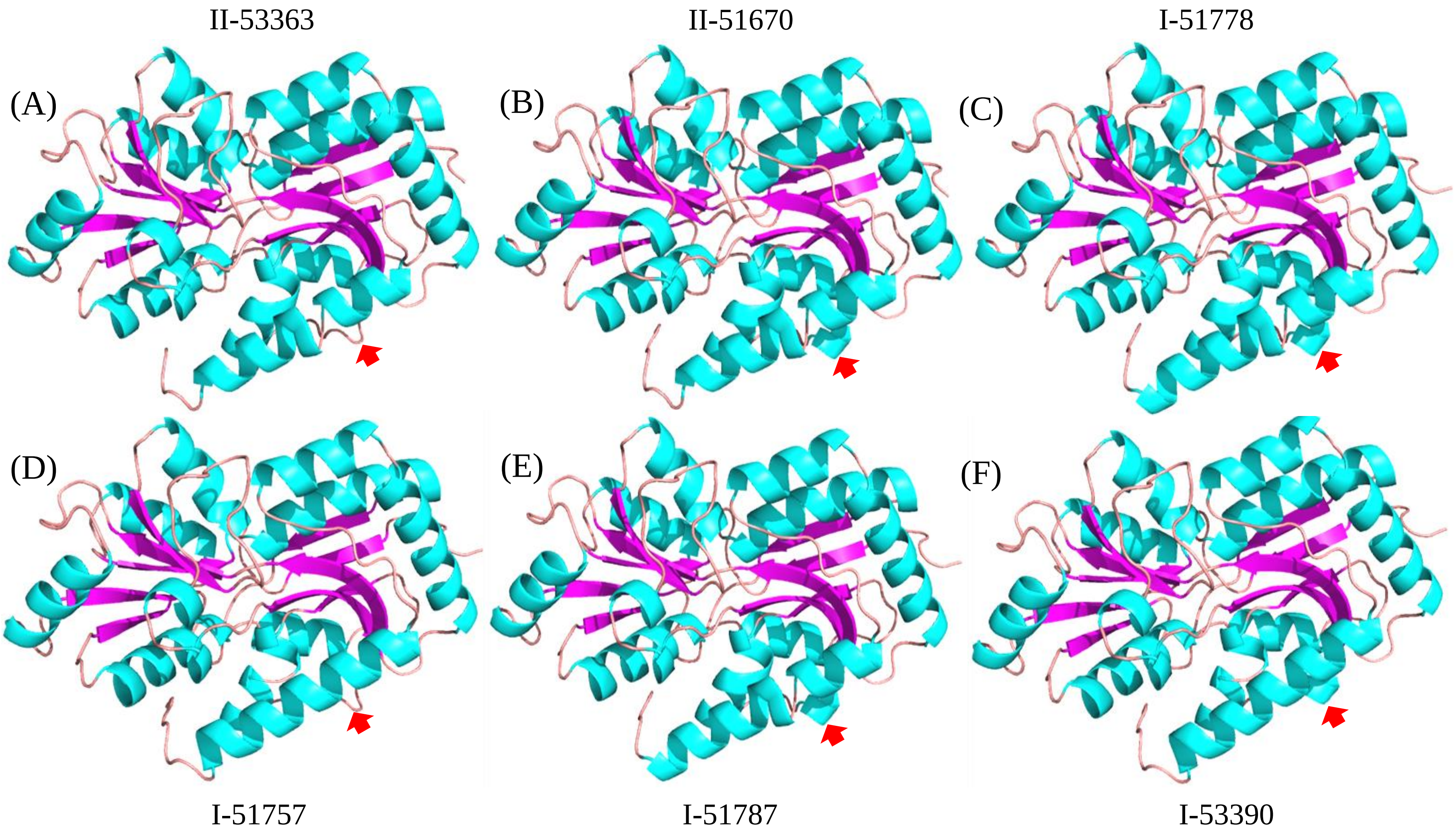

Supplementary Figure S4 Protein 3D structure for L373 proteins in Type II strains CCBAU 53363 (A) and CCBAU 51670 (B); and Type I strains CCBAU 51778 (C), CCBAU 51757 (D), CCBAU 51787 (E) and CCBAU 53390 (F) using SWISS-MODEL server and Pymol software.

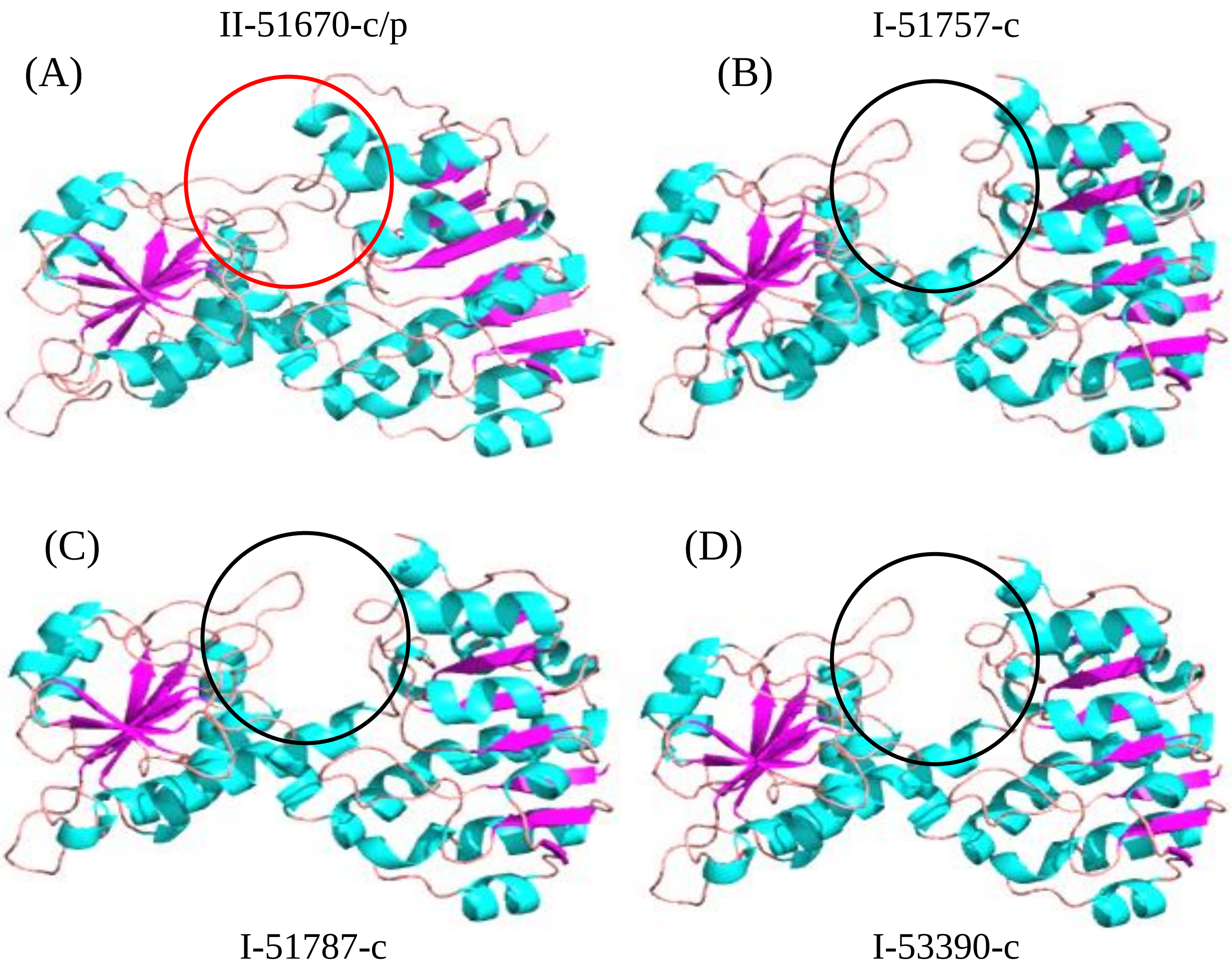

Supplementary Figure S5 Protein Subunit 3D structure predictions for L147 gene in Type II strains CCBAU 51670 chromosome and plasmid (A); Type I strains CCBAU 51757 chromosome (B), CCBAU 51787 chromosome (C) and CCBAU 53390 chromosome (D) using SWISS-MODEL server and Pymol software.

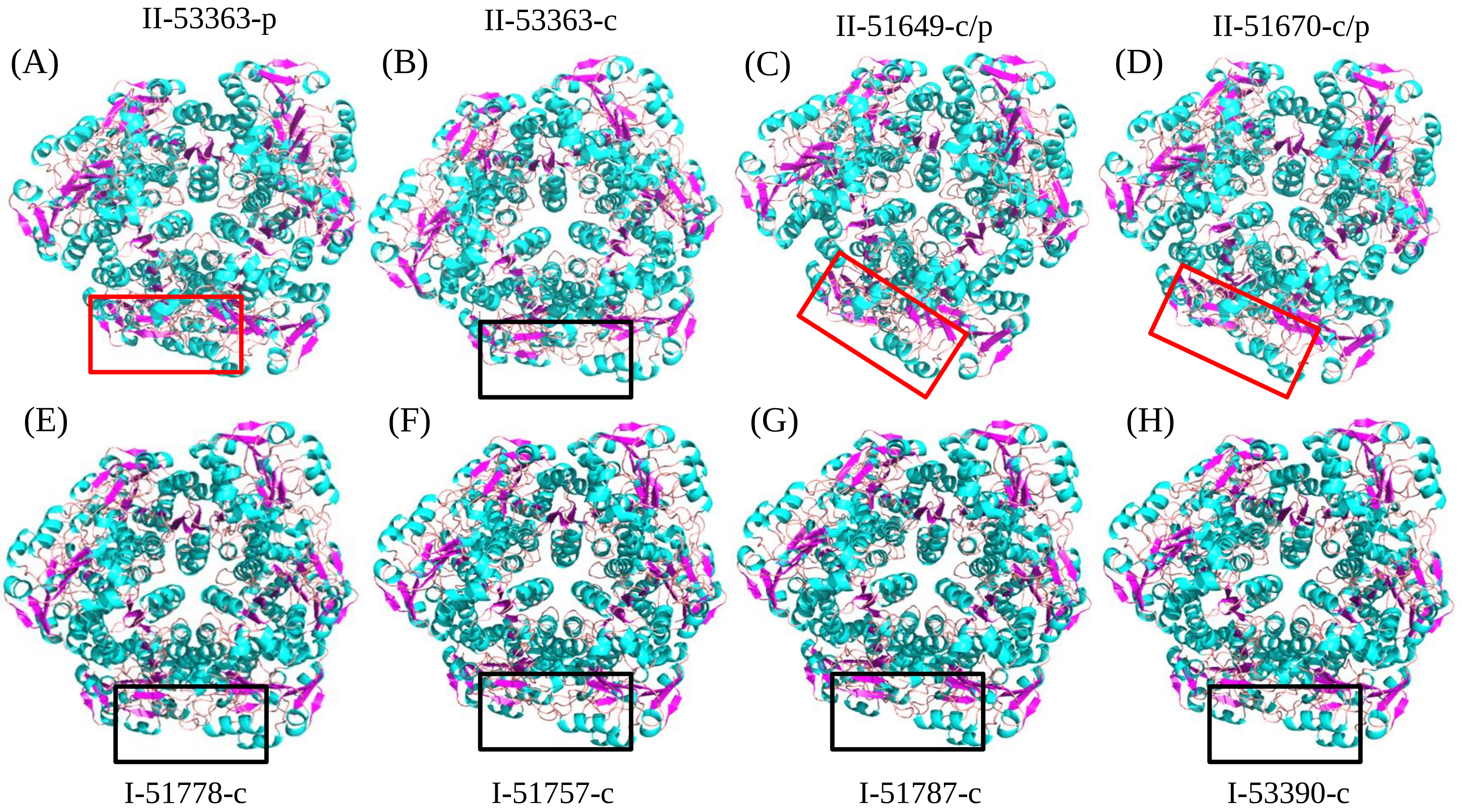

Supplementary Figure S6 Hexamer protein 3D structure predictions for L147 gene in Type II strains CCBAU 53363 plasmid (A), CCBAU 53363 chromosome (B), CCBAU 51649 chromosome and plasmid (C), CCBAU 51670 chromosome and plasmid (D); Type I strains CCBAU 51778 chromosome (E), CCBAU 51757 chromosome (F), CCBAU 51787 chromosome (G) and CCBAU 53390 chromosome (H) using SWISS-MODEL server and Pymol software.
